# Clinical outcomes in patients with Philadelphia chromosome-positive leukemia treated with ponatinib in routine clinical practice—data from a Belgian registry

**DOI:** 10.1007/s00277-021-04507-x

**Published:** 2021-05-04

**Authors:** Timothy Devos, Violaine Havelange, Koen Theunissen, Stef Meers, Fleur Samantha Benghiat, Alain Gadisseur, Gaëtan Vanstraelen, Hélène Vellemans, Benjamin Bailly, Nikki Granacher, Philippe Lewalle, Ann De Becker, Koen Van Eygen, Mia Janssen, Agnes Triffet, Inge Vrelust, Dries Deeren, Dominiek Mazure, Julie Bekaert, Michael Beck, Dominik Selleslag

**Affiliations:** 1 Department of Hematology, University Hospitals Leuven and Department of Microbiology and Immunology, Laboratory of Molecular Immunology (Rega Institute), KU Leuven, Campus Gasthuisberg, Herestraat 49, B-3000 Leuven, Belgium; 2 UCL Saint-Luc, Woluwe-Saint-Lambert, Belgium; 3 Jessa Ziekenhuis, Hasselt, Belgium; 4 Algemeen Ziekenhuis Klina, Brasschaat, Belgium; 5 Hôpital Erasme, Bruxelles, Belgium; 6 Universitair Ziekenhuis Antwerpen, Edegem, Belgium; 7 CHR Verviers, Verviers, Belgium; 8 CHU UCL Namur, Site Godinne, Yvoir, Belgium; 9 Hôpital de Jolimont, Haine-Saint-Paul, Belgium; 10 Ziekenhuis Netwerk Antwerpen Stuivenberg, Antwerpen, Belgium; 11 Institut Jules Bordet, Université Libre de Bruxelles, Bruxelles, Belgium; 12 Universitair Ziekenhuis Brussel, Jette, Belgium; 13 Algemeen Ziekenhuis Groeninge, Kortrijk, Belgium; 14 Ziekenhuis Oost-Limburg, Genk, Belgium; 15 Centre Hospitalier Universitaire Charleroi Vésale, Charleroi, Belgium; 16 Algemeen Ziekenhuis Sint-Elisabeth, Turnhout, Belgium; 17 Algemeen Ziekenhuis Delta, Roeselare, Belgium; 18 Universitair Ziekenhuis Gent, Gent, Belgium; 19 Incyte Biosciences International sàrl, Morges, Switzerland; 20 Incyte Biosciences Benelux B.V., Amsterdam, The Netherlands; 21 Algemeen Ziekenhuis Sint-Jan Brugge, Brugge, Belgium

**Keywords:** Ponatinib, Routine clinical practice, Registry, Chronic myeloid leukemia, Philadelphia chromosome-positive acute lymphoblastic leukemia

## Abstract

Data on clinical use of ponatinib are limited. This prospective registry aimed to evaluate outcomes of ponatinib treatment in routine practice over 3 years (2016–2019) in Belgium (NCT03678454). Patients with chronic myeloid leukemia (CML) or Philadelphia chromosome-positive (Ph+) acute lymphoblastic leukemia (ALL) were treated with ponatinib per current label. Fifty patients (33 CML and 17 Ph+ ALL) were enrolled. Fifty-five percent of CML and 29% of Ph+ ALL patients had received ≥3 prior tyrosine kinase inhibitors (TKIs). Reasons for starting ponatinib were intolerance (40%), relapse or refractoriness (28%) to previous TKIs, progression (16%), or *T315I* mutation (16%). Median follow-up was 15 months for CML and 4.5 months for Ph+ ALL patients. Best response was a major molecular response in 58% of CML and 41% of Ph+ ALL patients. Of 20 patients who started ponatinib due to intolerance to previous TKIs, 9 (64%) CML and 4 (67%) Ph+ ALL achieved a major molecular response. Three-year estimates of overall survival were 85.3% and 85.6%, respectively, in CML and Ph+ ALL patients; estimated progression-free survival was 81.6% and 48.9%. Adverse reactions were reported in 34 patients (68%); rash (26%) and dry skin (10%) were most common. Reported cardiovascular adverse reactions included vascular stenosis (3), arterial hypertension (2), chest pain (1), palpitations (1), and vascular occlusion (1). This Belgian registry confirms results from the PACE clinical trial and supports routine ponatinib use in CML and Ph+ ALL patients who are resistant or intolerant to previous TKIs or with the *T315I* mutation.

**Supplementary Information:**

The online version contains supplementary material available at 10.1007/s00277-021-04507-x.

## Introduction

Treatment with tyrosine kinase inhibitors (TKIs) has led to a major improvement in the prognosis of patients with chronic myeloid leukemia (CML) and Philadelphia chromosome-positive (Ph+) acute lymphoblastic leukemia (ALL) [1]. TKIs selectively target the constitutively active BCR-ABL1 tyrosine kinase, therefore suppressing the growth of malignant cells [2]. One of the challenges in TKI therapy is the development of resistance, in many cases through the presence or acquisition of mutations in the ABL kinase domain [2]. In addition, some patients become intolerant to TKI treatment by developing adverse events (AEs) that cannot always be managed through dose reductions or symptomatic treatment [3]. These AEs are a real challenge in the daily practice of hematologists.

Currently, several TKIs are used in clinical practice, i.e., imatinib, nilotinib, dasatinib, bosutinib, and ponatinib [4]. Ponatinib is a third-generation TKI with potent activity against native and mutated BCR-ABL1 containing single point mutations, including the *T315I* mutation, for which no other marketed TKI is effective [5, 6]. Ponatinib received marketing approval based on the results of the phase 2 **P**onatinib Ph+ **A**LL and **C**ML **E**valuation (PACE) trial, which demonstrated the efficacy and safety of ponatinib in patients with CML or Ph+ ALL who were intolerant or resistant to previous lines of TKI therapy [7, 8]. By 12 months, 56% of chronic phase (CP)-CML patients achieved a major cytogenetic response (MCyR; primary endpoint) [7]. The 5-year follow-up data of this trial showed deep and durable responses to ponatinib therapy in CP-CML patients [8]. In total, 60% of the evaluable CP-CML patients achieved MCyR, and the probability of maintaining MCyR for 5 years was 82%. Fifty-four percent of evaluable CP-CML patients achieved a complete cytogenetic response (CCyR) and 40% achieved a major molecular response (MMR, defined as *BCR-ABL1* mRNA levels ≤0.1% on the International Scale). Ponatinib dose reductions were proactively implemented during the course of the trial to decrease the risk of arterial occlusive events (AOEs). The response to therapy was maintained 40 months after ponatinib dose reduction by ≥90% of patients who had achieved MCyR or MMR. Five-year estimated rates of progression-free survival (PFS) and overall survival (OS) were 53% and 73%, respectively [8]. In Ph+ ALL patients, the efficacy data demonstrated promising results, with 41% of patients having a major hematologic response, 47% MCyR, and 38% CCyR [8].

The most common treatment-emergent AEs reported in the PACE trial were rash, abdominal pain, thrombocytopenia, headache, dry skin, and constipation. AOEs received special attention in the clinical trial, as they can provoke dramatic consequences for the patients. The cumulative incidence of AOEs in all patients was 17.1% (11.8% serious AOE) in the initial report and 25% (20% serious AOE) in the 5-year follow-up of the PACE trial. The risk for AOE incidence appeared to be related to ponatinib dose and pre-existing cardiovascular risk factors [7, 8].

Despite ponatinib’s authorization in Europe since 2013 [9], data on the use of ponatinib in routine clinical practice are scarce. Nevertheless, collection of such data is important to evaluate the effectiveness and safety of ponatinib and may help optimize the use of ponatinib in daily practice. This registry is, to our knowledge, the first report of prospective data of patients with CML or Ph+ ALL treated with ponatinib in routine clinical practice.

## Methods

### Registry design and patients

**Fig. 1:**
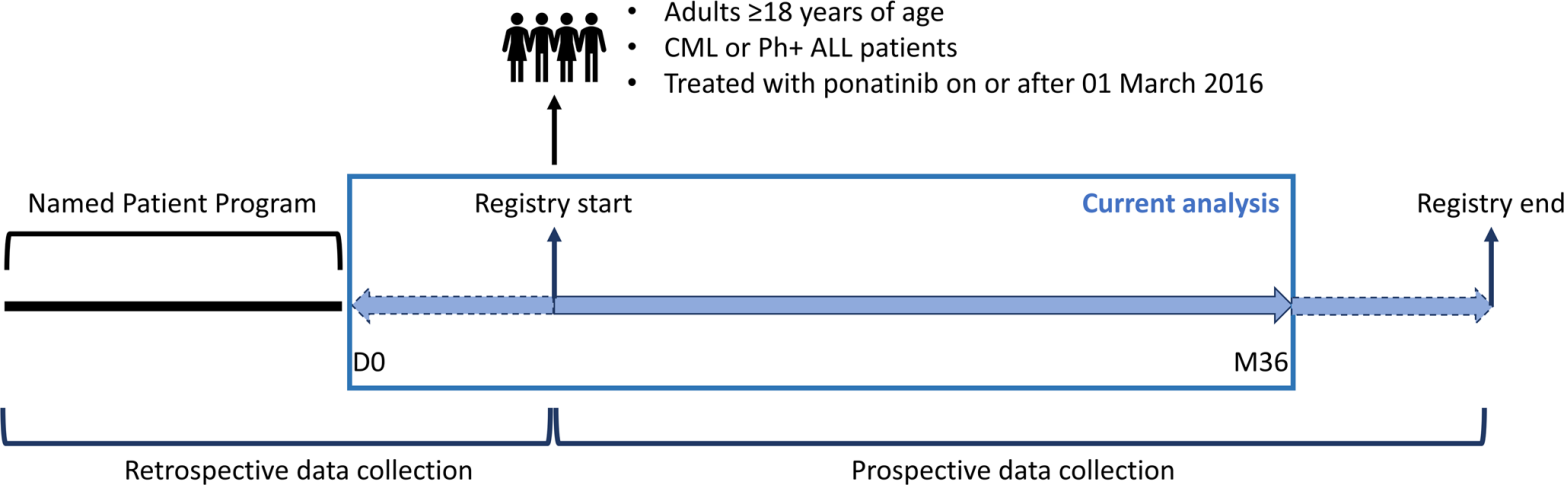
Study design. D, day; M, month; CML, chronic myeloid leukemia; Ph+ ALL, Philadelphia chromosome-positive acute lymphoblastic leukemia; D0, the start of reimbursement of ponatinib in Belgium, i.e., March 1, 2016

This multi-center, prospective, observational study is currently ongoing at 20 centers throughout Belgium (clinicaltrials.gov identifier: NCT03678454). Patients ≥18 years old, diagnosed with CML (all phases) or Ph+ ALL, and who started ponatinib treatment according to the indication in the product label [9] between March 1, 2016, the date of reimbursement in Belgium, and March 1, 2019, were eligible for this enrolment period. In addition, patients, who had participated in a named patient program (NPP) for ponatinib prior to reimbursement but were still being treated with ponatinib on March 1, 2016, were also allowed to participate. Patients were excluded if they participated in a clinical trial at any time during the registry period (Fig. 1).

This registry was conducted in accordance with the Declaration of Helsinki and the International Council for Harmonization guidelines for good clinical practice. All patients provided written informed consent before data collection, but a waiver was granted by the ethics committee (EC) for NPP patients who were still on ponatinib treatment on March 1, 2016, but deceased before the registry start. The registry protocol and the informed consent form were approved by the central EC and local ECs at each participating center; any subsequent amendments were approved by the central EC and notified to local ECs.

All procedures related to registry conduct, including data management, statistical analysis, and scientific writing, were performed by Modis, Wavre, Belgium.

### Data collection

All data used in the current analysis were collected as of treatment initiation. Data from registry start (which occurred approximately 10 months after reimbursement date) until month 36 were collected prospectively. As treatment initiation could be earlier than registry start, data between March 1, 2016 (date of reimbursement) and registry start were collected retrospectively. For the 8 patients treated in the NPP and still on treatment at registry start, data that had been collected in the NPP were transferred to the database for this registry. Safety data were not collected during the NPP. However, for NPP patients who participated in the registry, safety data were also collected retrospectively between March 1, 2016 and registry start and prospectively as of registry start.

Data were collected during routine treatment visits in the hospital or at least every 6 months. Documentation and date of informed consent, participation in NPP, patient demographics, disease characteristics, medical history, treatment patterns, treatment outcomes, and safety were collected. All AEs had to be reported based on a pre-defined AE list (Supplementary Table S1), with the option to describe any AE not included in the list, and were followed up until the outcome of the event was established. For all AEs, a possible causal relationship to ponatinib was assessed by the investigator. Responses to treatment for each patient were measured as per routine clinical practice and were defined according to the European LeukemiaNet 2013 recommendations [10]. Mutation testing was done as per routine clinical practice [10].

### Data analysis

Data were analyzed using descriptive statistics. OS and PFS curves were generated using the Kaplan–Meier method. The sample size was not pre-defined. The primary analysis was performed on the overall population. To identify any potential selection bias due to inclusion of patients who had participated to the NPP, a secondary analysis was performed excluding these patients. Subgroup analyses were also performed by disease (Ph+ ALL versus CML), and within the disease for those who started ponatinib due to intolerance or another reason than intolerance (i.e., progression, relapse, refractoriness, or *T315I* mutation). Subgroup analyses were also performed for patients who underwent an allogeneic stem cell transplantation before starting ponatinib treatment and for patients with MMR as best response to ponatinib. A sensitivity analysis was performed to investigate a potential imbalance in the CML patient population due to the inclusion of advanced phase CML patients.

## Results

### Patient characteristics

A total of 33 patients with CML and 17 with Ph+ ALL were included in this registry (Table 1). Among the CML patients, 30 were in chronic phase, 1 in accelerated phase, 1 in blastic myeloid phase, and 1 in blastic lymphoid phase. Out of 11 CML and 10 Ph+ ALL patients with mutations in the *BCR-ABL1* kinase domain, 6 (55%) CML and 6 (60%) Ph+ ALL patients had the *T315I* mutation. No mutations were detected in 19 (58%) patients with CML and 7 (41%) patients with Ph+ ALL, and mutation status was not determined for 3 (9%) patients with CML. Median age was 58 (19–83) years for CML and 56 (28–80) years for Ph+ ALL patients (Table 1). In total, 39% of CML and 35% of Ph+ ALL patients had a history of cardiovascular disease, 33% of CML and 35% of Ph+ ALL patients presented with arterial hypertension, 15% of CML and 18% of Ph+ ALL patients had diabetes mellitus, 12% of CML and 6% of Ph+ ALL patients had hyperlipidemia, and 30% of CML and 18% of Ph+ ALL patients were smokers. One patient with Ph+ ALL had a history of heart failure.

**Table 1:** Patient baseline characteristics

	All patients (*N* = 50)	CML patients (*N* = 33)	Ph+ ALL patients (*N* = 17)
Age in years, median (range)	58 (19–83)	58 (19–83)	56 (28–80)
Age in years, mean (SD)	56.2 (15.56)	56.8 (15.62)	55.1 (15.86)
Female, *n* (%)	19 (38%)	12 (36%)	7 (41%)
Previous TKI lines, *n* (%)			
1 TKI	4 (8%)	3 (9%)	1 (6%)
2 TKIs	23 (46%)	12 (36%)	11 (65%)
≥3 TKIs	23 (46%)	18 (55%)	5 (29%)
Presence of mutations, *n* (%)			
*T315I*	12 (24%)	6 (18%)	6 (35%)
Other	10 (20%)	5 (15%)	5 (29%)^a^
Not determined	3 (6%)	3 (9%)	-
Medical history, *n* (%)			
Liver disorder	2 (4%)	-	2 (12%)
Pancreas disorder	2 (4%)	2 (6%)	-
Reduced kidney function	6 (12%)	2 (6%)	4 (24%)
Hypertension	17 (34%)	11 (33%)	6 (35%)
Cardiovascular disease	19 (38%)	13 (39%)	6 (35%)
Smoking	13 (26%)	10 (30%)	3 (18%)
Diabetes	8 (16%)	5 (15%)	3 (18%)
Hyperlipidemia	5 (10%)	4 (12%)	1 (6%)
Hypercholesterolemia	6 (12%)	2 (6%)	4 (24%)
Significant alcohol abuse	4 (8%)	4 (12%)	-
Other	36 (72%)	21 (64%)	15 (88%)

*CML* chronic myeloid leukemia; *Ph+ ALL* Philadelphia chromosome-positive acute lymphoblastic leukemia; *TKI* tyrosine kinase inhibitor; *SD* standard deviation; *N* total number of patients; *n* number of patients in respective category

^a^In total, there were 10 Ph+ ALL patients with mutations. One patient had a *T315I* mutation and another mutation

### Previous treatment

Among patients with CML, 3 (9%) received 1 previous line of TKI treatment (all with *T315I* mutation), 12 (36%) received 2 previous lines, and 18 (55%) received 3 or more previous lines (Table 1). The corresponding numbers among Ph+ ALL patients were 1 (6%), 11 (65%), and 5 (29%), respectively. None of the Ph+ ALL patients had received more than 3 lines of different TKIs. Imatinib was the most commonly used first-line TKI, being used by 22 (67%) CML and 16 (94%) Ph+ ALL patients, while dasatinib was the most common second-line TKI, used in 19 (58%) CML and 14 (82%) Ph+ ALL patients. Nilotinib was used as a third-line therapy in 7 (21%) CML and 4 (24%) Ph+ ALL patients, and bosutinib as a fourth-line therapy in 4 (12%) CML patients. Two (6%) patients with CML and 6 (35%) patients with Ph+ ALL had received an allogeneic stem cell transplantation prior to ponatinib treatment.

### Ponatinib treatment

**Fig. 2:**
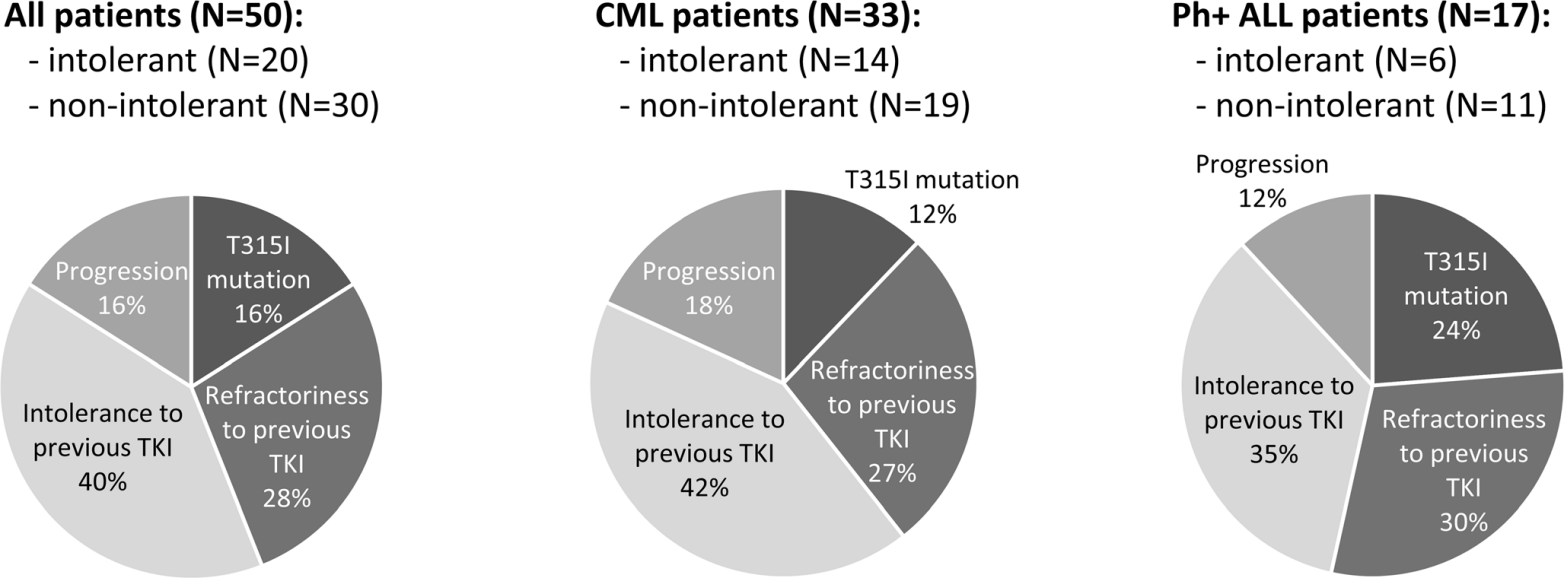
Reasons for starting ponatinib treatment. CML, chronic myeloid leukemia; Ph+ ALL, Philadelphia chromosome-positive acute lymphoblastic leukemia; intolerant, those who started ponatinib due to intolerance to previous tyrosine kinase inhibitors (TKIs); non-intolerant, those who started ponatinib due to reasons other than intolerance to previous TKIs (progression, *T315I* mutation, relapse or refractoriness). Refractoriness to previous TKI in this figure included absence of response, primary refractoriness, and hematological or cytogenetic relapse on previous TKI. Percentages may not add up to 100 due to rounding

Treatment with ponatinib was started in 14 patients with CML due to intolerance to previous TKI (42%), in 6 patients due to progression on previous TKI (18%), in 9 patients due to relapse or refractoriness (absence of response, primary refractoriness, hematological or cytogenetic relapse) to previous TKI (27%), and in 4 patients due to the *T315I* mutation (12%). Among Ph+ ALL patients, reasons to start treatment with ponatinib were intolerance to previous TKI (6, 35%), disease progression (2, 12%), relapse or refractoriness (absence of response, primary refractoriness, hematological or cytogenetic relapse [5, 30%]), or the *T315I* mutation (4, 24%) (Fig. 2).

The time from CML or Ph+ ALL diagnosis and the start of ponatinib treatment varied, ranging from 44 to 8139 days for CML patients and from 158 to 3632 days for Ph+ ALL patients. The majority of patients (70% of CML and 76% of Ph+ ALL) received ponatinib at the starting dose of 45 mg/day. The starting dose of 30 mg/day was used in 12% of patients with CML and in 12% of patients with Ph+ ALL, and the starting dose of 15 mg/day was used in 15% of patients with CML and in 12% of patients with Ph+ ALL. One patient with CML started the therapy with 15 mg of ponatinib every other day. There was a difference over time in the proportion of CML patients receiving the registered 45 mg/day starting dose of ponatinib: Among the first 10 patients included in the registry, 86% of CML patients received a starting dose of 45 mg/day, while in the last 10 included patients, only 43% of CML patients started with 45 mg/day.

### Treatment outcomes

**Fig. 3:**
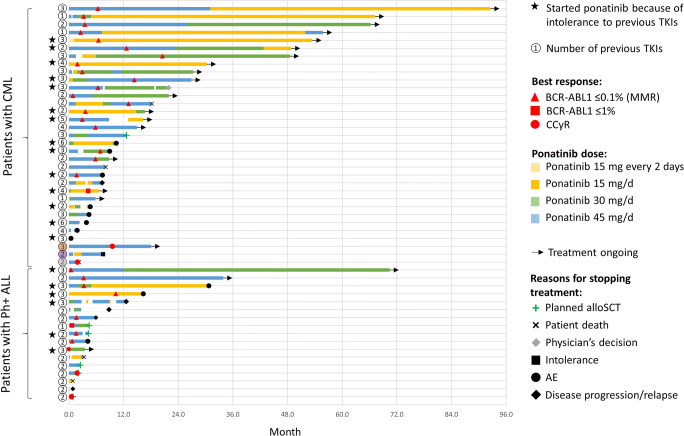
Swimmer plot displaying treatment duration, treatment modification, and outcome for each patient. Each bar represents an individual patient. Patients with CML were in chronic phase, except for 3 indicated patients: orange circle, blastic myeloid phase; purple circle, accelerated phase; grey circle, blastic lymphoid phase; AE, adverse event; CCyR, complete cytogenetic response; MMR, major molecular response; alloSCT, allogeneic stem cell transplantation; TKI, tyrosine kinase inhibitor

The median follow-up was 449 days (range, 15–2777) and 135 days (range, 26–2114) for patients with CML and Ph+ ALL, respectively. The median duration of ponatinib treatment was 380 days (range, 15–2777) for CML patients and 123 days (range, 13–2114) for Ph+ ALL patients. A swimmer plot detailing treatment duration and outcomes and of all individual patients in the registry is presented in Fig. 3.

**Fig. 4:**
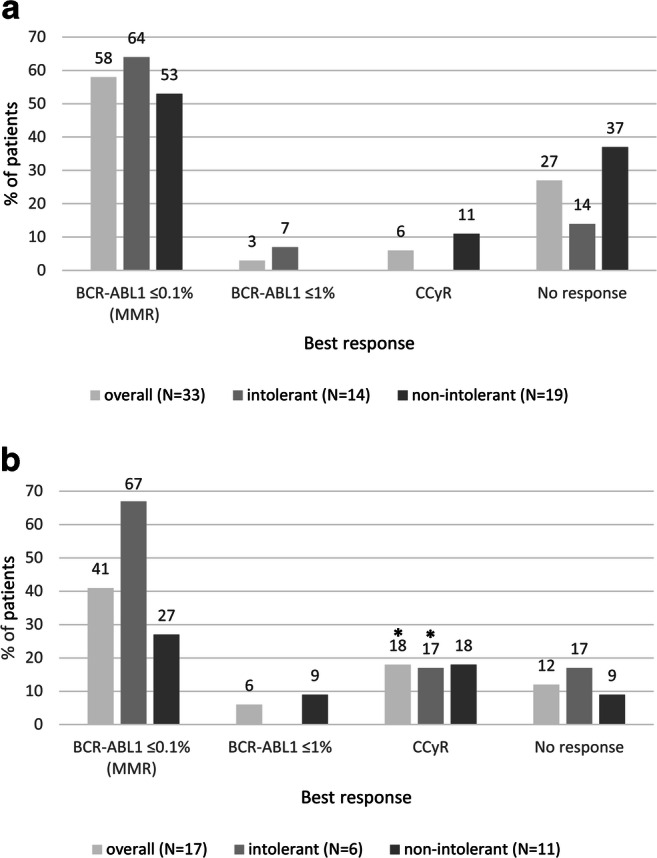
Best response to treatment in patients with **a** CML and **b** Ph+ ALL (overall, intolerant and non-intolerant patients). CCyR, complete cytogenetic response; CML, chronic myeloid leukemia; MMR, major molecular response; N, number of patients; Ph+ ALL, Philadelphia chromosome-positive acute lymphoblastic leukemia. *1 patient had achieved CCyR before starting treatment with ponatinib. Of note, 2 (6%) patients with CML and 4 (24%) patients with Ph+ ALL did not have measurable responses to treatment

MMR (*BCR-ABL1* mRNA ≤0.1%) was achieved as best response by 26 patients: 19 (58%) patients with CML and 7 (41%) patients with Ph+ ALL (Fig. 4). Of the CML patients who started with ponatinib at 45 mg/day, 30 mg/day, and 15 mg/day, respectively, 15 out of 23 (65%), none out of 4 and 3 out of 5 (60%) achieved MMR as best response. Also, the only CML patient starting at 15 mg every other day achieved MMR. Of the Ph+ ALL patients who started with 45 mg/day, 30 mg/day, and 15 mg/day, respectively, 6 out of 13 (46%), none out of 2 and 1 out of 2 (50%) achieved MMR as best response. Two (6%) patients with CML and 3 (18%) patients with Ph+ ALL achieved CCyR as best response (including 1 patient with Ph+ ALL who had CCyR before starting ponatinib treatment) and 1 patient from each group (3% and 6% of CML and Ph+ ALL patients, correspondingly) achieved *BCR-ABL1* mRNA ≤1% as best response. Nine (27%) patients with CML and 2 (12%) patients with Ph+ ALL did not achieve any response. The median time to best response was 151 days (range, 26–616) for patients with CML and 49 days (range, 14–308) for patients with Ph+ ALL. Among the 20 patients who started ponatinib because of intolerance to previous TKIs, MMR was achieved as best response by 9 (64%) CML and 4 (67%) Ph+ ALL patients and 1 (7%) CML patient achieved *BCR-ABL1* mRNA ≤1% (Fig. 4). The median time to best response in patients who started ponatinib due to previous TKI intolerance was 159 days (range, 51–431) in patients with CML and 73 days (range, 14–308) in patients with Ph+ ALL. Among the 30 patients who started ponatinib for reasons other than intolerance to previous TKI(s), 10 (53%) patients with CML and 3 (27%) patients with Ph+ ALL achieved MMR as best response. The median time to best response in these patients was 140 days (range, 26–616) and 36 days (range, 19–97), respectively. A sensitivity analysis, excluding the 3 CML patients in accelerated or blastic phase, did not show any difference in the percentage of CML patients who started ponatinib due to intolerance and who achieved MMR as best response compared to those who did not show intolerance with MMR as best response.

**Fig. 5:**
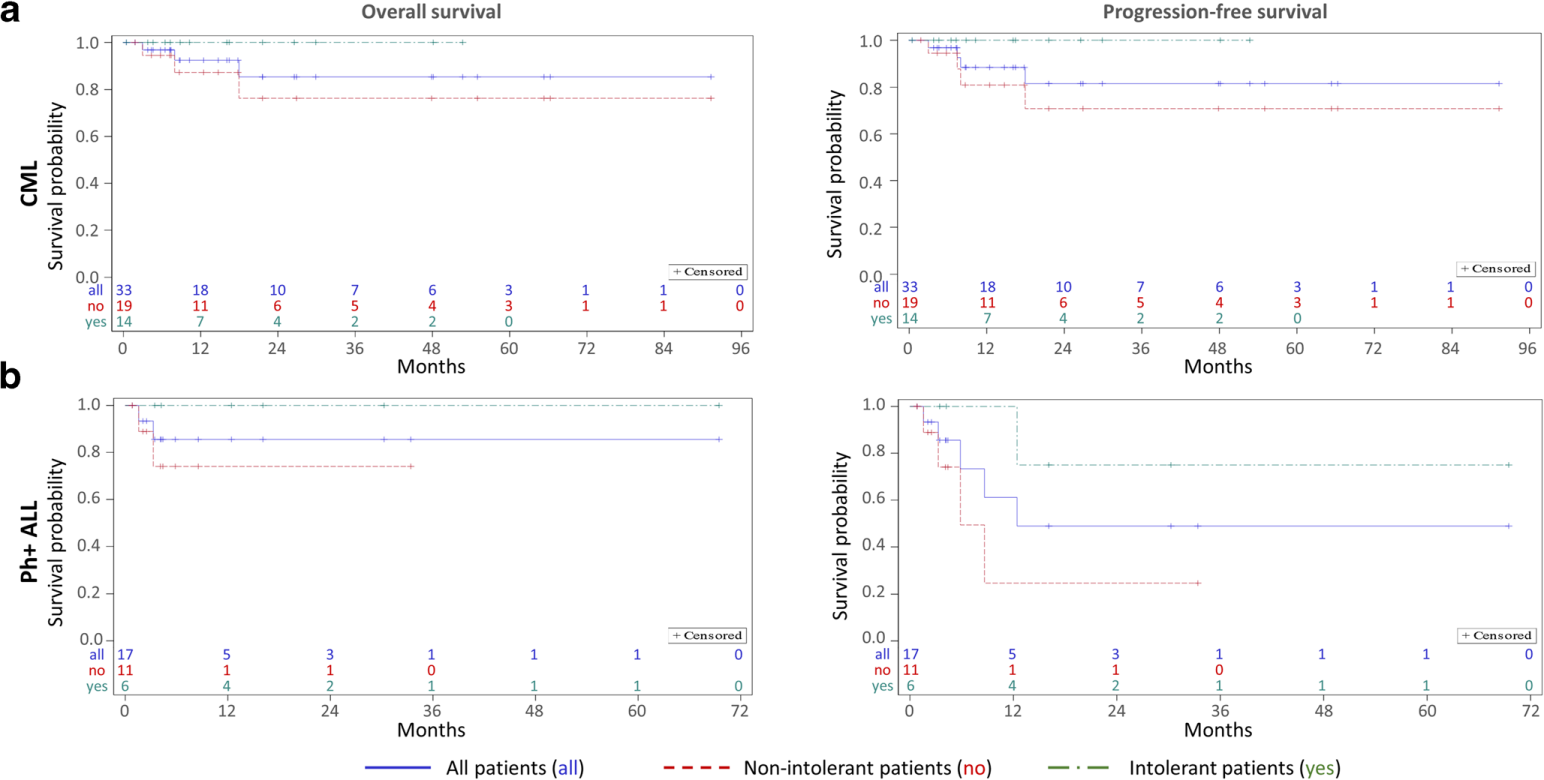
Kaplan-Meier estimates of overall survival and progression-free survival in **a** patients with CML (overall, intolerant and non-intolerant patients) and **b** patients with Ph+ ALL (overall, intolerant and non-intolerant patients). CML, chronic myeloid leukemia; Ph+ ALL, Philadelphia chromosome-positive acute lymphoblastic leukemia; TKI, tyrosine kinase inhibitor; OS, overall survival; PFS, progression-free survival

Estimated OS was 85.3% for CML and 85.6% for Ph+ ALL patients over 3 years of the registry duration. Estimated PFS was 81.6% for CML and 48.9% for Ph+ ALL patients (Fig. 5).

Dose reductions occurred in 20 (61%) CML and 7 (41%) Ph+ ALL patients, and dose increases occurred in 14 (42%) and 3 (18%) patients with CML and Ph+ ALL, respectively (Table 2). Dose reductions or interruptions occurred mostly due to AEs, but also to prevent future AEs as part of a risk management strategy in CML and Ph+ ALL patients. Reasons for dose increases in CML patients and Ph+ ALL patients were absent or poor response and good tolerance to a lower dose of ponatinib. Treatment interruptions were registered in 11 (33%) CML and 5 (29%) Ph+ ALL patients, with a median duration of interruption of 24 days (range, 7–126) in CML and 20 days (range, 12–201) in Ph+ ALL patients. In total, 15 CML (45%) and 14 (82%) Ph+ ALL patients terminated the treatment with ponatinib. Eight CML patients and 3 Ph+ ALL patients terminated treatment due to an AE.

**Table 2:** Overview of and reasons for treatment modifications

	All patients	CML patients	Ph+ ALL patients
Treatment modification, *n* (%)	*N* = 50	*N* = 33	*N* = 17
Dose reduction	27 (54)	20 (61)	7 (41)
Dose increase	17 (34)	14 (42)	3 (18)
Treatment interruption	16 (32)	11 (33)	5 (29)
Treatment termination	29 (58)	15 (45)	14 (82)
No change	5 (10)	3 (9)	2 (12)
Reasons for dose reduction/interruption, *n*’ (%)	*N*’ = 57	*N*’ = 38	*N*’ = 19
AE	42 (74)	29 (76)	13 (68)
Prevention	14 (25)	9 (24)	5 (26)
Other	1 (2)	-	1 (5)
Reasons for dose increase, *n*’ (%)	*N*’ = 24	*N*’ = 19	*N*’ = 5
No or low response	11 (46)	10 (53)	1 (20)
Good tolerance of treatment	13 (54)	9 (47)	4 (80)
Reasons for treatment termination, *n*’ (%)	*N*’ = 29	*N*’ = 15	*N*’ = 14
AE	11 (38)	8 (53)	3 (21)
Disease progression	4 (14)	1 (7)	3 (21)
Intolerance	1 (3)	1 (7)	-
Planned allogeneic stem cell transplantation	6 (21)	1 (7)	5 (36)
Other ^a^	7 (27)	4 (27)	3 (21)

*AE* adverse event; *N* total number of patients; *N’* total number of treatment modifications (dose increase, dose decrease/interruption or treatment termination); *n (%)* number (percentage) of patients in respective category; *n’ (%)* number (percentage) of treatment modifications in respective category

^a^This includes the 5 deaths in the registry

In general, results in the subgroup of patients who underwent an allogeneic stem cell transplantation before starting ponatinib were in line with the overall population. Of the 8 patients treated in the NPP who were included in this registry, 6 patients are currently still on treatment and achieved MMR. These patients have been on ponatinib treatment from 4 up to 7 years.

### Safety

**Fig. 6:**
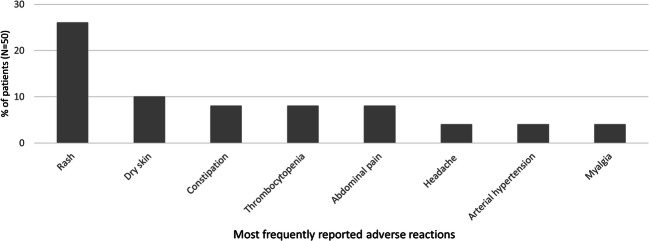
The most frequently reported adverse reactions. N, number of patients

Thirty-four (68%) patients experienced adverse reactions. The most frequently reported adverse reactions (by ≥10% of patients) were rash and dry skin (Fig. 6). Other reported adverse reactions of interest included thrombocytopenia (4), abdominal pain (4), vascular stenosis (3), arterial hypertension (2), chest pain (1), palpitations (1), vascular occlusion (1), pancytopenia (1), increased serum lipase (1), cholecystitis (1), hepatitis (1), cholestasis (1), pneumonia (1), hyponatremia (1), pancreatitis (1), and hepatocellular injury (1).

In total, 5 deaths were registered; none of these was considered by the investigator to be a consequence of the ponatinib treatment. Three patients suffered from general physical health deterioration resulting in death and two patients died from cardiorespiratory arrest induced by euthanasia (one patient with relapse of Ph+ ALL; one patient with both metastatic renal cell carcinoma and relapse of Ph+ ALL).

## Discussion

This registry collected data on the use of ponatinib in patients with CML or Ph+ ALL in routine clinical practice in Belgium. Data were collected in 20 hospitals that covered most of the patient population treated with ponatinib in the country. The results of this registry are in line with those of the PACE clinical trial [7, 8], although differences in patient population, size, study design, and follow-up hinder direct comparison. The median follow-up in this registry was 15 months for patients with CML and 4.5 months for patients with Ph+ ALL. A longer follow-up will be possible since this registry will continue for another 3 years. The number of patients harboring mutations in this registry was in line with PACE [8]. The overall percentage of patients with CML and Ph+ ALL who achieved MMR as best response was 58% and 41%, respectively. It should be noted that information on patients’ responses to any previous TKI (before starting the registry) was not collected. This limits the interpretation of the treatment responses as we do not know whether responses occurred rapidly after starting ponatinib or whether responses from previous treatment were maintained. For CP-CML patients, the duration of response in the registry was in line with the duration seen in the PACE trial. More than one-third of all patients (42% CML and 35% Ph+ ALL patients) started ponatinib treatment due to intolerance to previous TKIs.

Cross-intolerance to different TKIs is generally not considered to be clinically relevant, particularly for non-hematologic AEs [11]. While 20 patients (14 CML and 6 Ph+ ALL) initially started ponatinib because of intolerance to previous TKIs, only 1 patient in this registry discontinued ponatinib treatment due to intolerance. This was a CML patient who had switched to ponatinib for another reason than intolerance. From the 20 patients who initially started ponatinib because of intolerance to previous TKIs, 11 discontinued ponatinib treatment, of which 8 due to an AE (73%). From the 30 patients who started ponatinib for other reasons than intolerance, 18 discontinued ponatinib treatment, of which 3 due to an AE (17%). Patients who discontinued due either to an AE or to treatment intolerance were considered to be intolerant in this analysis. It confirms that, even if some patients are intolerant to multiple TKIs, ponatinib can still be a valuable option for intolerant patients. While all patients in PACE initially started ponatinib at a dose of 45 mg/day, dose reductions were recommended subsequently in CP-CML patients who had achieved MCyR or MMR to reduce the risk for AOEs [8]. Most of those patients maintained their responses despite dose reductions. Based on the currently approved label, the starting dose of ponatinib is 45 mg/day. However, some patients in this registry received a lower starting dose. In the few patients who started with 15mg/day, this starting dose did not seem to preclude achieving MMR. A trend towards reduction of ponatinib starting dose in CML patients was observed during the course of this registry, as reflected by the proportion of patients receiving the maximal starting dose among the first and the last 10 patients enrolled in the registry. However, these results do not allow us to make any conclusions in this regard due to the small number of patients receiving each dose and current study design. While the recently updated recommendations from the European LeukemiaNet recommend a lower starting dose for certain CML patients [5], more data from randomized clinical trials are needed to further evaluate this. The effect of different starting doses of ponatinib (15 mg/day, 30 mg/day, and 45 mg/day) on the occurrence of AEs and on efficacy in CML patients is currently being evaluated in the prospective dose-ranging trial OPTIC (Optimizing Ponatinib Treatment in CML, clinicaltrials.gov identifier: NCT02467270).

Safety outcomes were similar to those observed in the PACE trial for the most frequently observed AEs. The incidence of cardiovascular AEs seemed low in this observational registry (reported for 8 patients [16%]), even though patient baseline characteristics might indicate certain risk factors for cardiovascular events. In the PACE trial, 31% of CP-CML and 25% of Ph+ ALL patients reported treatment-emergent AOEs of any grade. This difference could be due to the appropriate patient selection and monitoring, the implementation of the risk minimization activities, and a possible underreporting of AEs in a real-life situation as compared to a clinical trial or shorter follow-up period; this difference was also observed for the frequency of other AEs. The Common Terminology Criteria for Adverse Events grading was not collected for the purpose of this registry, limiting the clinical interpretation (severity) of the described AEs.

Several retrospective observational studies reporting clinical use of ponatinib have been published [12–14]. However, to the best of our knowledge, this Belgian registry is the first to prospectively evaluate the effectiveness and safety of ponatinib in routine clinical practice. This nationwide registry collected quality data from leading hospitals that treat CML and Ph+ ALL patients in Belgium. Although the number of patients included in the registry was slightly higher compared to published retrospective studies, the available number of patients per diagnosis was still too small to allow robust statistical analysis. Hence, all analyses in this registry were descriptive.

In conclusion, the results obtained in routine clinical practice in Belgium are in line with those of the PACE trial, therefore supporting the use of ponatinib in patients with CML and Ph+ ALL after failure or intolerance to previous lines of TKIs or who have the *T315I* mutation. Major molecular responses were observed as best response in most CML patients and a large proportion of Ph+ ALL patients. In Ph+ ALL patients, deep molecular responses seemed to occur more frequently in intolerant versus non-intolerant patients. The registry revealed no new safety signals other than those previously reported [7, 8]. The collection of registry data is still ongoing, with the aim of evaluating more patients and allowing for longer follow-up.

## Supplementary information

ESM 1 (DOCX 22 kb)

## Data Availability

The datasets generated during and/or analyzed during the current study are available from the corresponding author on reasonable request.
