# Supplementary material for: Clinical outcomes in patients with Philadelphia chromosome-positive leukemia treated with ponatinib in routine clinical practice—data from a Belgian registry
Source: Ann Hematol. 2021 May 4;100(7):1723–32. doi: 10.1007/s00277-021-04507-x (PMC8195783; doi:10.1007/s00277-021-04507-x)
Supplement: Supplementary file 1 — (DOCX 22 kb) [file 277_2021_4507_MOESM1_ESM.docx]

**Real-world outcomes in patients with Philadelphia chromosome-positive leukemia treated with ponatinib – Data from a Belgian registry**

**Annals of Hematology**

**Authors:**

Timothy Devos,^1^ Violaine Havelange,^2^ Koen Theunissen,^3^ Stef Meers,^4^ Fleur Samantha Benghiat,^5^ Alain Gadisseur,^6^ Gaëtan Vanstraelen,^7^ Hélène Vellemans,^8^ Benjamin Bailly,^9^ Nikki Granacher,^10^ Philippe Lewalle,^11^ Ann De Becker,^12^ Koen Van Eygen,^13^ Mia Janssen,^14^ Agnes Triffet,^15^ Inge Vrelust,^16^ Dries Deeren,^17^ Dominiek Mazure,^18^ Julie Bekaert,^19#^ Michael Beck^20^ and Dominik Selleslag^21^

**Affiliations:**

^1^Department of Hematology, University Hospitals Leuven and Department of Microbiology and Immunology, Laboratory of Molecular Immunology (Rega Institute), KU Leuven, Leuven, Belgium

^2^UCL Saint-Luc, Woluwe-Saint-Lambert, Belgium

^3^Jessa Ziekenhuis, Hasselt, Belgium

^4^Algemeen Ziekenhuis Klina, Brasschaat, Belgium

^5^Hôpital Erasme, Bruxelles, Belgium

^6^Universitair Ziekenhuis Antwerpen, Edegem, Belgium

^7^CHR Verviers, Verviers, Belgium

^8^CHU UCL Namur, Site Godinne, Yvoir, Belgium

^9^Hôpital de Jolimont, Haine-Saint-Paul, Belgium

^10^Ziekenhuis Netwerk Antwerpen Stuivenberg, Antwerpen, Belgium

^11^Institut Jules Bordet, Université Libre de Bruxelles, Bruxelles, Belgium

^12^Universitair Ziekenhuis Brussel, Jette, Belgium

^13^Algemeen Ziekenhuis Groeninge, Kortrijk, Belgium

^14^Ziekenhuis Oost-Limburg, Genk, Belgium

^15^Centre Hospitalier Universitaire Charleroi Vésale, Charleroi, Belgium

^16^Algemeen Ziekenhuis Sint-Elisabeth, Turnhout, Belgium

^17^Algemeen Ziekenhuis Delta, Roeselare, Belgium

^18^Universitair Ziekenhuis Gent, Gent, Belgium

^19^Incyte Biosciences International sàrl, Lausanne, Switzerland

^20^Incyte Biosciences Benelux B.V., Amsterdam, The Netherlands

^21^Algemeen Ziekenhuis Sint-Jan Brugge, Brugge, Belgium

^#^Current affiliation: Incyte Biosciences International sàrl, Morges, Switzerland

**Corresponding author:**

Prof. Dr. Timothy Devos, MD

Hematology department

University Hospitals Leuven (UZ Leuven)

Campus Gasthuisberg

Herestraat 49

B-3000 Leuven, Belgium

Email: [timothy.devos@uzleuven.be](mailto:timothy.devos@uzleuven.be)

Phone: +32 16 34 68 80

**Supplementary Table S1. Predefined list of adverse events**

| **Adverse event** |
| --- |
| Abdominal pain  Anaemia  Arthralgia  Atrial fibrillation  Cardiac failure  Cerebrovascular accident  Constipation  Decreased neutrophil count  Decreased platelet count  Diarrhoea  Dry skin  Dyspnoea  Fatigue  Febrile neutropenia  Headache  Hypertension  Increased lipase  Myalgia  Myocardial infarction  Nausea  Pain in back  Pain in extremity  Pancreatitis  Pancytopenia  Pericardial effusion  Pneumonia  Pyrexia  Rash  Thrombocytopenia  Transient ischemic attack  Other |
